# Cell Cycle Abnormalities Associated with Differential Perturbations of the Human U5 snRNP Associated U5-200kD RNA Helicase

**DOI:** 10.1371/journal.pone.0062125

**Published:** 2013-04-29

**Authors:** Ali Ehsani, Jessica V. Alluin, John J. Rossi

**Affiliations:** Department of Molecular and Cellular Biology, and Irell and Manella Graduate School of Biological Sciences, Beckman Research Institute of the City of Hope, Duarte, California, United States of America; Florida State University, United States of America

## Abstract

Splicing of pre-messenger RNAs into functional messages requires a concerted assembly of proteins and small RNAs that identify the splice junctions and facilitate cleavage of exon-intron boundaries and ligation of exons. One of the key steps in the splicing reaction is the recruitment of a tri-snRNP harboring the U5/U4/U6 snRNPs. The U5 snRNP is also required for both steps of splicing and exon-exon joining. One of the key components of the tri-snRNP is the U5 200kd helicase. The human U5-200kD gene isolated from Hela cells encodes a 200 kDa protein with putative RNA helicase function. Surprisingly, little is known about the functional role of this protein in humans. Therefore, we have investigated the role of the U5-200kD RNA helicase in mammalian cell culture. We created and expressed a dominant negative domain I mutant of the RNA helicase in HEK293 cells and used RNAi to downregulate expression of the endogenous protein. Transient and stable expression of the domain I mutant U5-200kD protein using an ecdysone-inducible system and transient expression of an anti-U5-200kD short hairpin RNA (shRNA) resulted in differential splicing and growth defects in the 293/EcR cells. Cell cycle analysis of the dominant negative clones revealed delayed exit from the G2/M phase of the cell cycle due to a mild splicing defect. In contrast to the domain I dominant negative mutant expressing cells, transient expression of an anti-U5-200kD shRNA resulted in a pronounced S phase arrest and a minute splicing defect. Collectively, this work demonstrates for the first time establishment of differential human cell culture splicing and cell cycle defect models due to perturbed levels of an essential core splicing factor.

## Introduction

Since the discovery that the coding information of eukaryotic genes is interrupted by introns [1], [2], the precision and complexity of intron removal from pre-mRNAs has been the subject of intense investigation. The large majority of human genes contain introns and most pre-mRNAs undergo alternative splicing. Therefore, it can be anticipated that perturbing the splicing process will have deleterious consequences on cell viability. Recently, Kittler et al. [3] reported that knockdown of several splicing factors in HeLa cells generated mitotic spindle defects and subsequent delays in cell division.

The spliceosome is comprised of four small ribonucleoproteins (snRNPs), U1, U2, U4/U6 and U5, as well as a large number of non-snRNP splicing factors. The number of specific proteins associated with each of the snRNPs varies. The most complex protein composition reported to date belongs to the 25S [U4/U6.U5] tri-snRNP complex [4]. The 20S U5 snRNP is composed of the highly structured U5 snRNA and eight specific proteins with molecular weights of 15, 40, 52, 100, 102, 116, 200 and 220 kDa [4]. It has been reported that the U5 specific 200 kD protein belongs to the DExH box family of putative RNA helicases [5]. The U5-200kD protein harbors one DEIH and one DEVH helicase domain [5]. These two domains also harbor all other sequence motifs required for helicase activity. To date, the U5-200kD is the only RNA helicase reported that contains two putative DExH helicase domains.

The yeast homologue of the U5-200kD, Prp44, (also called SNRNP200, ASCC3L1, HELIC2, Brr-2, Snu246p) is a 246 kDa protein that also possesses two DEXH-box RNA helicase domains [5]–[7]. There is a high degree of homology between the two proteins (43.6% identity; 64.2% similarity). However, the DExH domain I of the U5-200kD is more homologous to the yeast Prp44 domain I than its own DExH domain II. Unlike the U5-200kD, the yeast amino acid sequences of domain II are more degenerate [5]. It has been established that the yeast homologue is an intrinsic component of the yeast 25S [U4/U6.U5] tri-snRNP complex and that it is vital for cell viability [5], [8], [9]. Both *in vitro* and *in vivo* analyses of mutants in the helicase domain of this protein resulted in disruption of U4/U6 unwinding and decreased cell viability [5], [8], [9]. Antibody-mediated inhibition of U5-200kD function in HeLa cell nuclear splicing extracts demonstrated that this protein is involved in the second step of pre-mRNA splicing [5]. Additionally, purified U5 snRNP and the U5-200kD RNA helicase exhibited ATP-dependent U4/U6 RNA duplex unwinding *in vitro* [10].

To date, functional studies of the mammalian U5-200kD have been limited to biochemical analyses in cell and splicing extracts. Given this limitation, we set out to characterize the functional aspects of this protein in living cells. Two separate approaches have been experimentally tested: 1) use of a transiently and stably expressed helicase domain I DNT mutant in human HEK293 cells, and 2) si/shRNA mediated knockdown of the mRNA encoding the U5-200kD protein in HEK 293 cells. The most striking phenotypes observed using either approach were marked yet distinct cell cycle and splicing abnormalities, suggesting differential regulation of gene expression and therefore activation of different cell cycle checkpoints depending on the severity of the splicing defect. Our findings suggest a potential dual role of the helicase in splicing and cell cycle regulation.

## Results

### Expression of a Dominant Negative Mutant U5-200kD Protein in an Ecdysone-inducible 293/EcR Cell Line

In order to study the functional role of the U5-200kD protein in cell culture, we borrowed upon work conducted with the *S. cerevisiae* homologue [9] and created a dominant negative mutation in the first helicase domain of the human protein.

**Figure 1:**
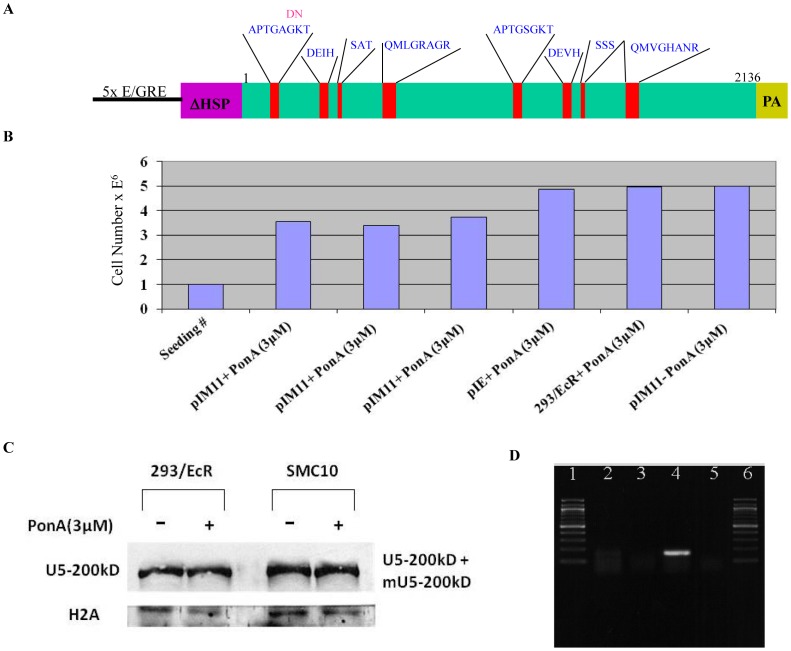
Expression analysis of the ecdysone-inducible mutant U5-200kD gene in HEK293 cells. A) *Organization of the regulatory elements in the ecdysone-inducible plasmid as well as the GKT-DNT inducible mutant U5-200kD, pI-M11.* Numbers 1-2136 indicate the amino acids in the ORF. PA is the polyadenylation site and ΔHSP is the minimal heat shock promoter on the ecdysone-inducible plasmid. B) *Transient expression of the inducible mutant U5-200kD gene.* The mutant U5-200kD gene (M11) cloned under the control of the ecdysone-inducible plasmid (pI-M11) was transfected into the trans-activator 293/EcR cell line clone (C13). The mutant gene was induced with 0.5, 1.0 and 3.0 mM of the inducer, Ponasterone A. For the controls, the enhanced green florescent protein (EGFP) was cloned under the inducible promoter and transfected into the C13 cell line (pIE-3.0 mM), 3.0 mM Ponasterone A was added to the C13 cell line (C13–3.0 mM) and the pIM11 plasmid was transfected into the C13 cell line without the inducer (pIM11-0 mM). The cell count analyses revealed growth retardation in the induced M11 dishes and not in the control plates. C) Western analysis of mutant U5-200KD protein expression. Histone 2A (H2A) was included as a loading control. D) *RT-PCR detection of the transiently transfected mutant U5-200kD gene.* Lanes 1 and 6 are the 100 bp ladders. Lane 2 is the RT-PCR reaction of the uninduced pI-M11 transfected into the 293/EcR cells. Lane 4 is the RT-PCR reaction of the induced pI-M11 transfected into the 293/EcR cells. Lanes 3 and 5 are the minus RT reactions for lanes 2 and 4, respectively.

Mutational analysis in yeast revealed that the mutation in the first helicase domain of the yeast homologue of U5-200kD (GKT to DNT) created a strong dominant negative mutant [9]. Additionally, it is well established that the amino acid sequence of domain I is highly conserved between yeast Prp44 and U5 200kD. We therefore chose the homologous sequence for generation of a GKT to DNT mutant in the human U5-200kD cDNA (Fig. 1A). The altered U5-200kD cDNA (hereafter referred to as M11) was sequenced in its entirety to verify that only the desired mutation was created (Fig. 1C).

Considering that ectopic expression of a dominant negative mutant U5-200kD might be deleterious, we attempted to establish an ecdysone-inducible expression system for the mutant. In order to accomplish this goal, we established HEK293 cell lines stably expressing the two trans-activating proteins required for induction of an insert cloned into the pIND plasmid. Three of our stably transfected HEK293/EcR clones were tested for the Ponasterone A-mediated induction of a pIND driven *Renilla reniformis* luciferase. Twenty-four hours post transfection of the luciferase reporter, Ponasterone A was added and twenty-four hours subsequent to induction the cells were harvested and assayed for luciferase expression. Clone #13 exhibited a 170 fold induction of luciferase (data not presented). A similar pattern of induction and expression of the luciferase gene, although not as strong, was observed for clone #1 (data not presented). Clone #13 was used for the U5-200kD dominant negative gene expression studies.

### Transient Transfection and Expression Analysis of the Inducible Mutant U5-200kD (pIND-M11) Gene in HEK293/ECR Cell Lines

**Figure 2:**
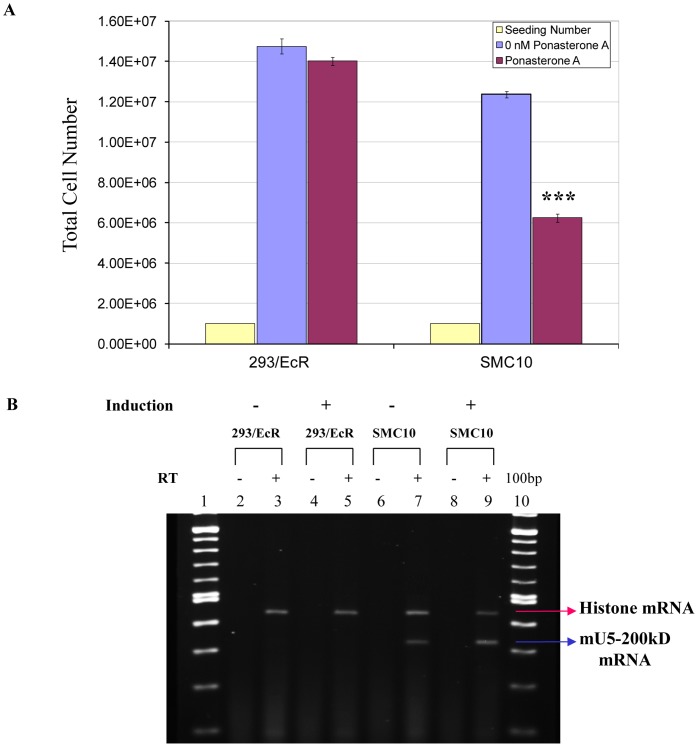
Stable expression of the mutant U5-200kD protein results in growth defects. A) *Growth defects in the Ponasterone A inducible, stably transfected mutant U5-200kD clones.* The uninduced and induced 293/EcR cells show a normal, wild type growth rate, indicating that the Ponasterone A had no effect on growth rate. p<0.001. B) *Detection of the mutant U5-200kD gene in SMC10 cells using mutant specific primers.* The mutant specific primers detected the mutant U5-200kD mRNA only in the SMC10 uninduced (lane 7 lower band) and induced (lane 9 lower band) cells. The top bands in lanes 3, 5, 7 and 9 are the histone PCR products. Lanes 2, 4, 6 and 8 are minus RT controls showing lack of DNA contamination. Lanes 1 and 10 are the 100 bp markers.

Equal concentrations of the ecdysone-inducible mutant M11 were transfected into replicate plates containing equal numbers of the HEK293/EcR cells (Fig. 1B). Expression of the M11 mutant was induced with various concentrations of Ponasterone A. As a control, pIND-EGFP harboring an ecdysone-inducible enhanced green fluorescent protein was also transfected into the HEK293/EcR cells. Additional controls included induced HEK 293/EcR cells and uninduced pIND-M11-transfected HEK293/EcR (Fig. 2B). The pIND-M11 transfected cells that were treated with various amounts of Ponasterone A exhibited similar levels of growth inhibition (Fig. 1B), whereas the uninduced pIND-M11 cells and other negative control cells did not show a proliferation defect (Fig. 1B).

Transient expression of the U5-200kD M11 mutant was confirmed via allele specific RT-PCR (Fig. 1D). Total RNA isolated from the uninduced pIND-M11 cells did not yield an RT-PCR product (Fig. 1D, lane 2). These data demonstrate the primer allele specificity since the endogenous U5-200kD transcript was not amplified.

The experiments with the M11 mutant suggested that expression of the dominant negative protein was responsible for the growth inhibitory phenotype. To examine this further, the pIND-M11 mutant was stably transfected into the HEK293/EcR cell line. Cells surviving G418 selection were diluted to obtain single clones and maintained under G418 selection. Cells from several of the clones were subjected to Ponasterone A induction, and their growth characteristics were compared with uninduced cells and mock transfected HEK 293/EcR cells. After three days of induction, cells were harvested and counted. Several of the clones grew more slowly than the controls even in the absence of the inducer Ponasterone A (data not presented). When these slow growing clones were treated with Ponasterone A, they grew even more slowly than their uninduced counterparts (Fig. 2A, data not shown). One of the clones (SMC10) exhibited the greatest growth defects. To verify the induction of the M11 allele, allele specific RT-PCR reactions were carried out on the RNAs isolated from the uninduced and induced SMC10 cells and the parental HEK293/EcR cells. Figure 2B shows that only the SMC10 cells expressed the M11 allele (Fig. 2B, lanes 7 and 9). Furthermore, once the M11 cDNA was integrated, there was leakiness in expression of the transcript, although the transcript level increased following addition of the inducer Ponasterone A (Fig. 2B, lanes 7 and 9).

### Analyses of Splicing Defects in the SMC10 Cell Line

**Figure 3:**
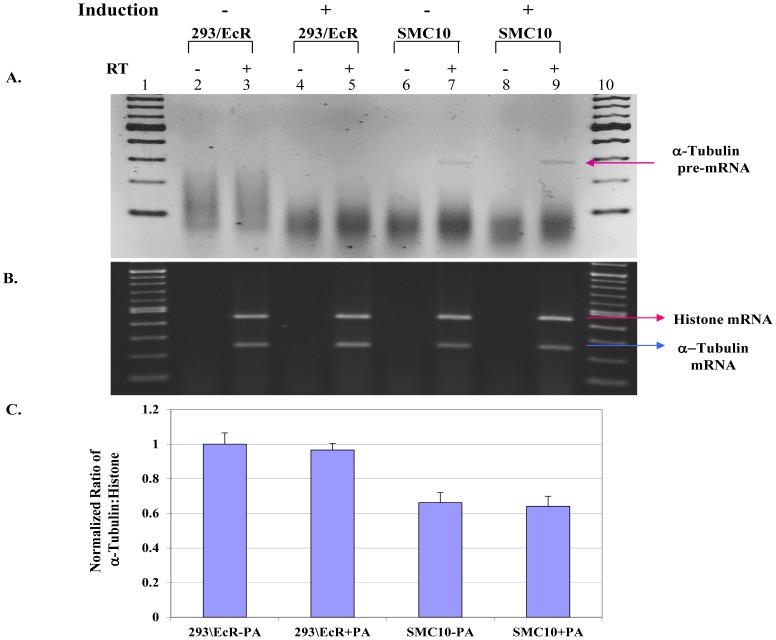
Stable expression of the mutant U5-200kD protein affected alpha-tubulin pre-mRNA splicing. A) *Detection of alpha-tubulin pre-mRNA.* Lanes 3, 5, 7 and 9 are RT-PCR reactions for detection of the alpha-tubulin pre-mRNA. Absent PCR product in lanes 3 and 5 demonstrate complete splicing of the endogenous alpha-tubulin pre-mRNA. The band in lanes 7 and 9 is the alpha-tubulin pre-mRNA that accumulated in the splicing-defective SMC10 cell lines. The stronger band for PCR products (lane 9) indicates increased accumulation of the pre-mRNA due to induced expression of the mutant U5-200kD protein. Lanes 1 and 10 are the 100 bp ladders. RT-PCR products were resolved in a 1.8% agarose gel and stained with ethidium bromide. B) *RT-PCR analysis of the alpha-tubulin mRNA.* The upper band in lanes 3, 5, 7 and 9 is the histone product amplified from the uninduced and induced 293/EcR and SMC10 RNAs, respectively. The lower band in those lanes is the alpha-tubulin amplified product. Lanes 2, 4, 6 and 8 are the minus reverse transcriptase reactions for each sample. Lanes 1 and 10 are the 100 bp ladders. Samples were resolved in a 1.8% agarose gel and stained with ethidium bromide. C) *Semi-quantitative analysis of the alpha-tubulin mRNA expression.* Compared to 293/EcR cells, the alpha-tubulin mRNA expression in the SMC10 cells has decreased approximately thirty-five percent. Induction of the mutant protein had little detectable effect on alpha-tubulin mRNA levels in the SMC10 clone.

We chose the alpha-tubulin mRNA as a target transcript to monitor potential splicing defects in SMC10 since it was previously demonstrated in yeast temperature sensitive mutants that yeast splicing factors *PRP16, PRP17* and *Cef1* impair alpha-tubulin splicing and are also defective in spindle assembly [11], [12]. Additionally, it has been reported that TPX2, a protein that is involved in chromosome induced microtubule assembly during spindle formation, co-purifies with spliceosome components [13], [14]. In the human genome there are six copies of the alpha-tubulin gene, which contains four exons and three introns. Initially, we used semi-quantitative RT-PCR to detect changes in the steady state levels of alpha-tubulin mRNA. HEK293/EcR and SMC10 cells were grown in the presence or absence of Ponasterone A. Total RNAs were isolated from these cell lines and subjected to semi-quantitative RT-PCR analysis using primers specifically designed to detect alpha-tubulin mRNA (3B lanes, 3, 5, 7 and 9). The amplification parameters were set within a linear, quantitative range. As an internal control, we used histone H2A, which lacks introns. Analyses of the RT-PCR products revealed an approximate thirty-five percent reduction in the alpha-tubulin mRNA level in the uninduced stable mutant clone 10 (Fig. 3C). The reduction of alpha-tubulin mRNA was slightly more in the induced than uninduced mutant cell culture, indicating that further induced expression of the mutant U5-200kD gene had only a modest additional effect on splicing of the alpha-tubulin mRNA. Considering that the reduction of alpha-tubulin mRNA in SMC10 cells could be due to a global transcriptional decrease and not a splicing defect, we designed primers to specifically detect the alpha-tubulin pre-mRNA. Total RNAs isolated from the induced and uninduced cells were analyzed by semi-quantitative RT-PCR (Fig. 3A). These analyses revealed the presence of unspliced alpha-tubulin RNA only in the SMC10 cells (Fig. 3A, lanes 7 and 9). The SMC10 cells treated with Ponasterone A had more alpha-tubulin precursor RNA than untreated SMC10 cells (Fig. 3A, lanes 7 and 9). We verified that the signal of the intron containing alpha-tubulin PCR product was not due to DNA contamination by performing the RT-PCR reactions in the presence and absence of reverse transcriptase (Fig. 3, lanes 2, 4, 6 and 8). Collectively, these data suggest that expression of the dominant negative U5-200kD protein resulted in only a mild perturbation of splicing as monitored by the accumulation of the alpha-tubulin pre-mRNA.

**Figure 4:**
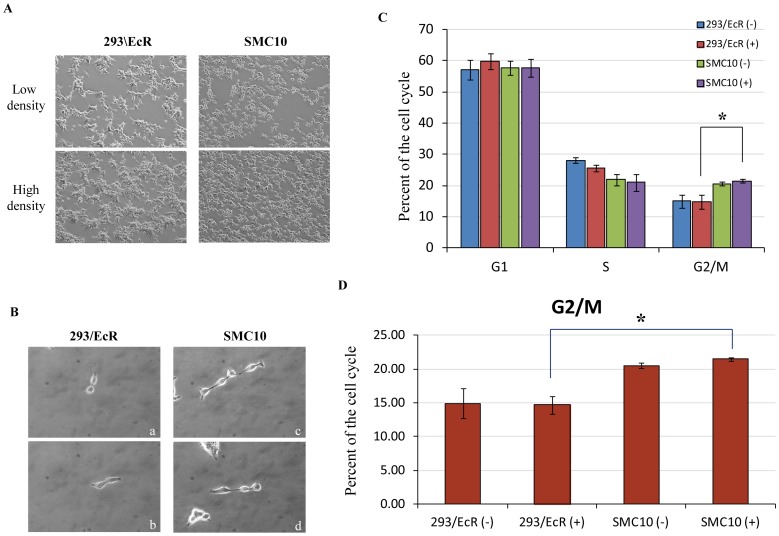
SMC10 cells have altered growth patterns and abnormal cell morphologies. A) *Growth pattern and size differences between the parental 293/EcR and stable mutant clone 10 cell lines.* B) *Comparisons of the growth morphologies of the 293/EcR and SMC10 cells.* a) 293/EcR cells undergoing cell division. b) 293/EcR cells prior to cytokinesis. c and d) SMC10 cells remain attached in dumbbell like structures. C) *Cell cycle analyses of the uninduced and Ponasterone A treated C13 and SMC10 cell lines.* In the SMC10 cells the number of cells in the G2/M phase has increased compared to the control 293/EcR cell lines. p<0.05. D) *Comparison of the G2/M and sub G1 phases of C13 and SMC10 cell lines.* The SMC10 cells are stalled at the G2/M phase. p<0.05.

Upon microscopic examination of the parental HEK293/EcR cells and SMC10 mutant expressing cells, we observed the latter to be markedly smaller in size (Fig. 4A). Also, the appearance of connected dumbbell-shaped cells in the induced SMC10 plates was striking (Fig. 4A, B). The observed growth defect and altered morphology of the SMC10 cells prompted us to investigate the cell cycle distribution of these and control cell lines. Whereas the cell cycle distribution of induced and uninduced HEK293/EcR cells appeared to be similar and mimicked HEK293 cells (Fig. 4C), in the SMC10 cell lines, the G1 phase decreased, and accordingly the G2/M phase increased significantly, whereas there was only a minor increase in the S phase (Fig. 4C). Separate analysis of the G2/M phase among the four cell populations revealed an increase in G2/M phase for both uninduced and induced SMC10 cells (Fig. 4D). These cell cycle analyses were performed twice on unsynchronized cells with identical results.

The G2/M phase of the cell cycle involves the nucleation and polymerization of tubulin monomers. Gamma-tubulin is used for nucleation, and alpha- and beta-tubulin heterodimers are used for the polymerization and formation of spindle fibers. The cell morphology (dumbbells) and cell cycle phenotype of the SMC10 cells indicates possible retardation of spindle fiber formation. This retardation is most likely due to the observed reduction of mature alpha-tubulin mRNA in the SMC10 cells.

### Downregulation of U5-200kD mRNA by Small Interfering RNAs

**Figure 5:**
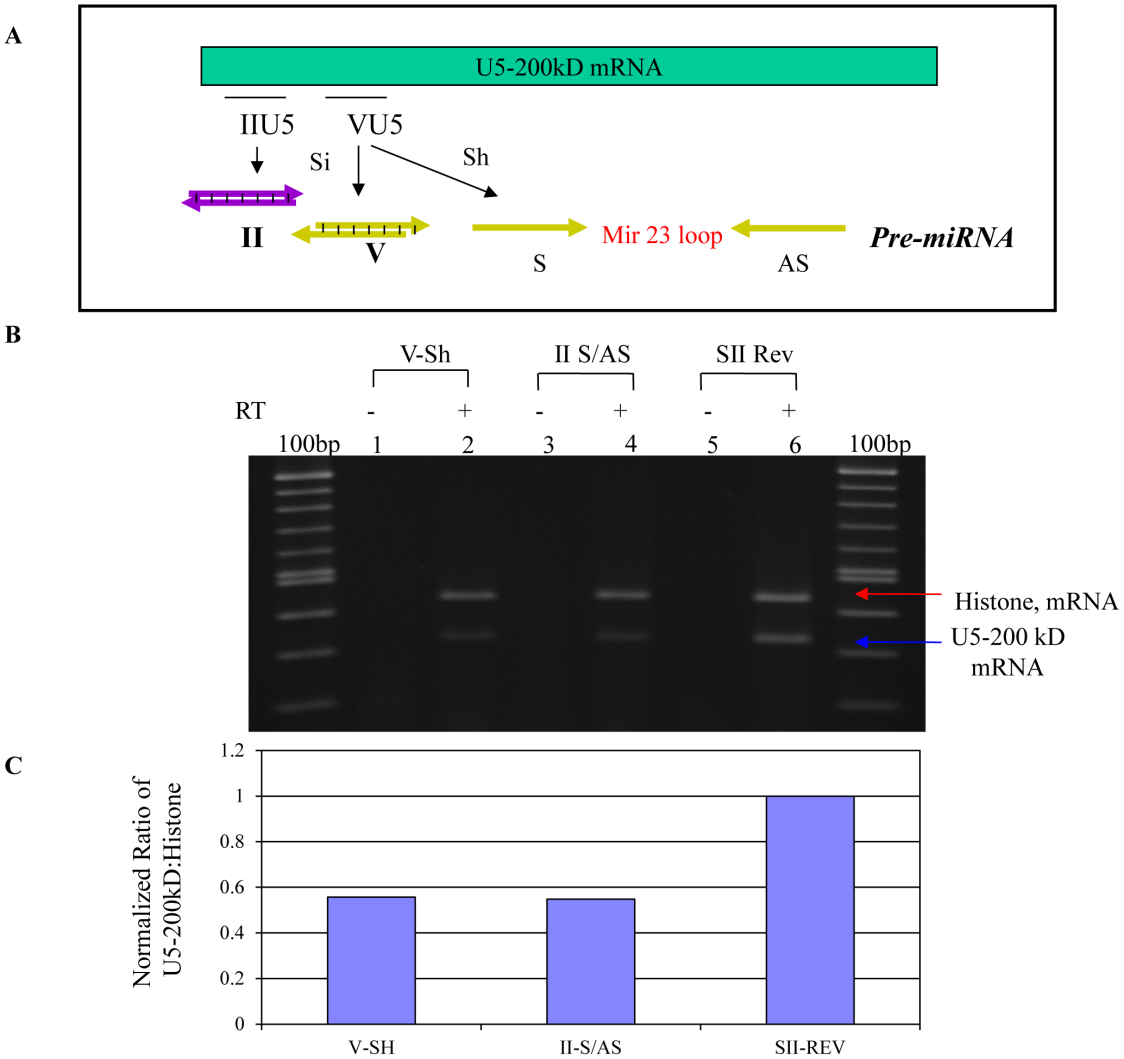
siRNA and shRNA knockdown of the U5-200kD mRNA. A) *Diagram of the site II siRNA and site V siRNA and shRNA targeting the N-terminal coding region of the U5-200kD mRNA.* B) *RT-PCR analyses of the 293/EcR cells transfected with anti-U5-200kD site II and V siRNAs and the irrelevant anti HIV-1 Rev siRNA.* The lower band in lanes 2, 4 and 6 is the RT-PCR amplified endogenous U5-200kD mRNA. The upper band is the RT-PCR amplified endogenous H2A which was used to normalize the expression of the U5-200kD mRNA. Lanes 1, 3 and 5 represent the RT-PCR reactions without addition of reverse-transcriptase. C) *Semi-quantitative analysis of the U5-200kD RT-PCR reactions.* After normalizing the U5-200kD signal to the histone signal, the bar graph for both site V and II treated cells indicate approximately forty-five percent reduction of the U5-200kD mRNA in the total isolated cellular RNA. Considering that the transfection efficiency for this experiment was approximately fifty percent, we assume close to one hundred percent reduction of the target mRNA in the transfected cells. Both sites V and II siRNAs were effective in reducing the U5-200kD mRNA in the 293/EcR cells.

**Figure 6:**
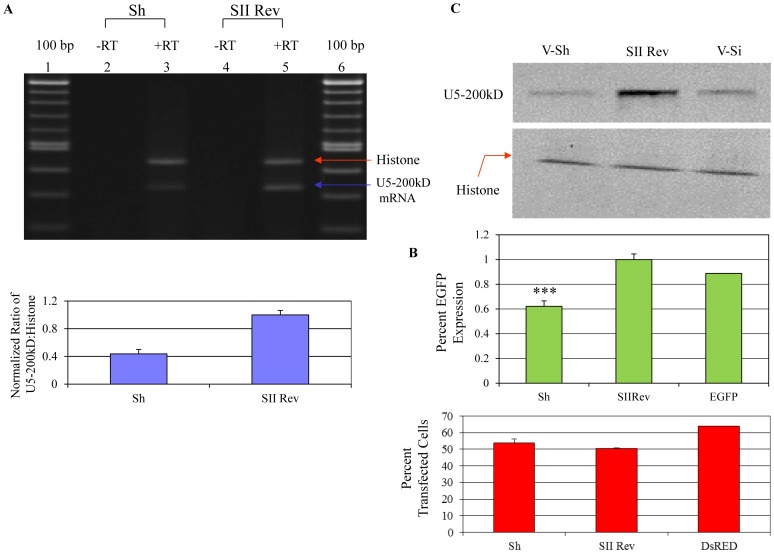
RT-PCR analysis of U5-200kD mRNA expression. A) (upper panel) Lanes 2 and 4 are the plus RT reactions indicating the expression of the histone mRNA (top band) and the U5-200kD mRNA (bottom band). Lane 2 is the RT-PCR product from the anti-U5-200kD shRNA treated cells and lane 4 is the RT-PCR analyses of the U5-200kD from control anti-Rev shRNA treated cells. Lanes 1 and 3 are the minus RT controls. (Lower panel) Semi-quantitative RT-PCR analysis of U5-200kD mRNA expression. The anti-U5-200kD shRNA effectively reduced the expression level of the target, U5-200kD mRNA. The irrelevant anti-Rev shRNA had no effect on the levels of the U5-200kD mRNA. C) Western Blot analyses of the U5-200kD and Histone 2A proteins. Targeting the U5-200kD mRNA by shRNA (lane 1) and siRNA (lane 3) resulted in reduction of the protein. The anti-Rev shRNA had no effect on the U5-200kD protein level (lane 2). Histone was included as a loading control. B) *FACS analyses of EGFP Expression.* (Upper panel) The processing of the int-EGFP reporter and therefore EGFP expression was reduced in cells treated with anti-U5-200kD shRNA. The irrelevant siRNA had no effect on the processing of the int-EGFP pre-mRNA. (Lower panel) *FACS analysis of transfection efficiency*. FACS analysis revealed comparable transfection efficiencies between the two treatments (anti-U5-200kD and site II Rev shRNAs). RFP expression was measured and the percent of red fluorescent cells reported. p<0.001.

In order to further investigate the role of the U5-200kD RNA helicase, we chose to use RNAi to knock down expression of the endogenous protein. Five siRNAs (expressed as separate sense and antisense RNAs) or short hairpin RNAs (shRNAs) targeting different sequences/regions of the U5-200kD mRNA were designed and cloned into the PCR2.1 plasmid as U6 promoter cassettes (Fig. 5A). Transfection of these constructs into both HEK293/EcR and the parental HEK293 cells demonstrated that separately expressed sense/antisense RNAs targeting site II, V, and a shRNA targeting site V were the most potent (Fig. 5B and C). As a negative control, we used an irrelevant short hairpin RNA targeting HIV rev RNA [15]–[17]. The mRNA knockdown results were confirmed by Western blotting for the U5-200kD protein (Fig. 6C).

To monitor the splicing defect created by downregulation of the endogenous U5-200kD helicase, we engineered a splicing reporter construct containing a small intron inserted upstream of the enhanced green fluorescent protein gene. The intron-EGFP gene was cloned into pIND so that the expression of the gene was controlled by the addition of Ponasterone A. The pDsRed2-N1 plasmid was included as a control for transfection efficiency in the experiments using the intron-EGFP reporter.

The plasmid encoding the site V shRNA was co-transfected with the EGFP splicing reporter and the DsRed2 plasmid into HEK293/EcR cells. Seventy-two hours post transfection Ponasterone A was added to the transfected cells. On the fourth day cells were initially examined microscopically and subsequently for the expression of EGFP using fluorescent activated cell sorting (FACS) analysis. The EGFP expression levels as well as RFP expression (for transfection efficiency) were measured. Expression of EGFP was reduced approximately thirty-eight percent in cells co-transfected with the site V short hairpin RNA compared to the irrelevant short hairpin and the reporter only transfected cells (Fig. 6B, upper panel). The transfection efficiencies were similar between the experimental samples (Fig. 6B, lower panel). These results indicated a decrease in splicing activity in the 293/EcR cells transfected with the anti-U5-200kD short hairpin and not with the irrelevant short hairpin RNA.

To confirm that the reduction in EGFP expression was due to a defect in splicing elicited by the shRNA, we carried out semi-quantitative RT-PCR analyses on the U5-200kD mRNA (Fig. 6A). The RT-PCR analyses revealed an approximate fifty-eight percent decrease in the level of U5-200kD mRNA (Fig. 6B, lanes 2 and 4). In the irrelevant short hairpin transfected cells, the normalized ratio of the U5-200kD to histone mRNA was similar to that observed with RNA from untreated cells. Western blot analyses of the U5-200kD protein revealed that both the short hairpin RNA targeting site V and the expressed S/AS siRNA targeting site V reduced the U5-200kD protein levels to the same extent (Fig. 6C).

**Figure 7:**
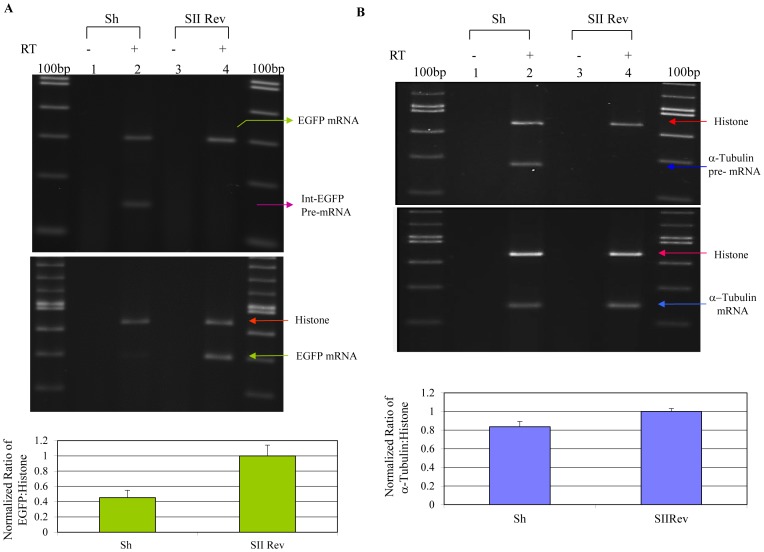
RT-PCR detection of EGFP pre-mRNA. A) (Upper panel) Lanes 2 and 4 illustrate detection of EGFP mRNA (upper band) and pre-mRNA (lower band) in RNA samples isolated from anti-U5-200kD shRNA and irrelevant shRNA treated cells, respectively. Lanes 1 and 3 are minus RT controls. (Middle panel) RT-PCR analysis of EGFP mRNA expression. Lanes two and four demonstrate expression of the histone (top band) and spliced EGFP mRNAs in anti-U5-200kD shRNA and irrelevant anti-EGFP rev shRNA treated 293/EcR cells. Lanes 1 and 3 are minus RT reactions. (Lower panel**)** Semi-quantitative RT-PCR analysis of the EGFP mRNA expression. B) *RT-PCR detection of alpha-tubulin pre-mRNA and histone mRNA.* (Upper panel) The lower band in Lane 2 is the alpha-tubulin pre-mRNA product from samples treated with U5-200kD specific shRNA and the irrelevant anti-Rev shRNA, respectively. The upper bands are the histone products used for normalization. Lanes 1 and 3 are minus RT controls. The 100bp size marker was used for product size verification. (Middle panel) RT-PCR detection of the alpha-tubulin and histone mRNAs. The lower bands in Lanes 2 and 4 are alpha-tubulin products from samples treated with the U5-200kD specific hairpin and the irrelevant anti-Rev shRNAs, respectively. The upper bands are the histone products used for normalization. Lanes 1 and 3 are minus RT controls. The 100bp size marker was used for product size verification. (Lower panel**)** Semi-quantitative analysis of alpha-tubulin mRNA expression. Semi-quantitative analysis of alpha-tubulin mRNA expression normalized against histone mRNA expression revealed only a 16% reduction in the expression of the alpha-tubulin mRNA in the splicing defect background.

To detect the splicing defect created by the site V short hairpin RNA and Site II S/AS RNA mediated downregulation of U5-200kD, expression of the EGFP mRNA was analyzed by semi-quantitative RT-PCR (Fig. 7A, middle panel, lanes 2 and 4, and Figure S1A). The RT-PCR data revealed the greatest decrease, approximately fifty-four percent, in the level of EGFP mRNA within the cells transfected with the U5-200kD specific short hairpin targeting site V (Fig. 7A, lower panel). Quantitative RT-PCR analysis of EGFP mRNA in site II S/AS siRNA transfected cells revealed reduction of EGFP mRNA compared to control shRNA transfected cells (Figure S1A bottom panel). The decrease in the level of EGFP mRNA (54%) corresponds to the knockdown level of the U5-200kD expression (58%). The level of the EGFP mRNA remained unchanged in the irrelevant short hairpin transfected cells.

As a corollary to the measurements of EGFP mRNAs, we also analyzed the accumulation of intron containing transcripts in the site V shRNA and site II S/AS siRNA transfected cells versus control treated cells. RT-PCR analyses using pre-mRNA specific primers showed accumulation of the intron containing EGFP only in cells treated with the site V shRNA (Fig. 7A, upper panel) and the site II S/AS siRNA (Figure S1A). Since the alpha-tubulin mRNA in the dominant negative mutant expressing cells decreased approximately thirty five percent, we investigated whether the splicing defect due to reduction of the U5-200kD also affected alpha-tubulin expression. A semi-quantitative alpha-tubulin specific RT-PCR analysis was performed using RNAs prepared from cells expressing the U5-200kD specific site V shRNA, site II S/AS siRNA and the irrelevant shRNAs (Fig. 7B, middle panel, and Figure S1B). Surprisingly, and in contrast to the M11 results, these analyses revealed moderate downregulation in the alpha-tubulin mRNA levels (∼16%) as a consequence of downregulation of the U5-200kD message (Fig. 7B, lower panel), although after extensive PCR amplification (two rounds) we did detect some accumulation of the alpha-tubulin intron containing transcripts in the shRNA site V and site II treated samples (Fig. 7B, upper panel and Figure S1B).

**Figure 8:**
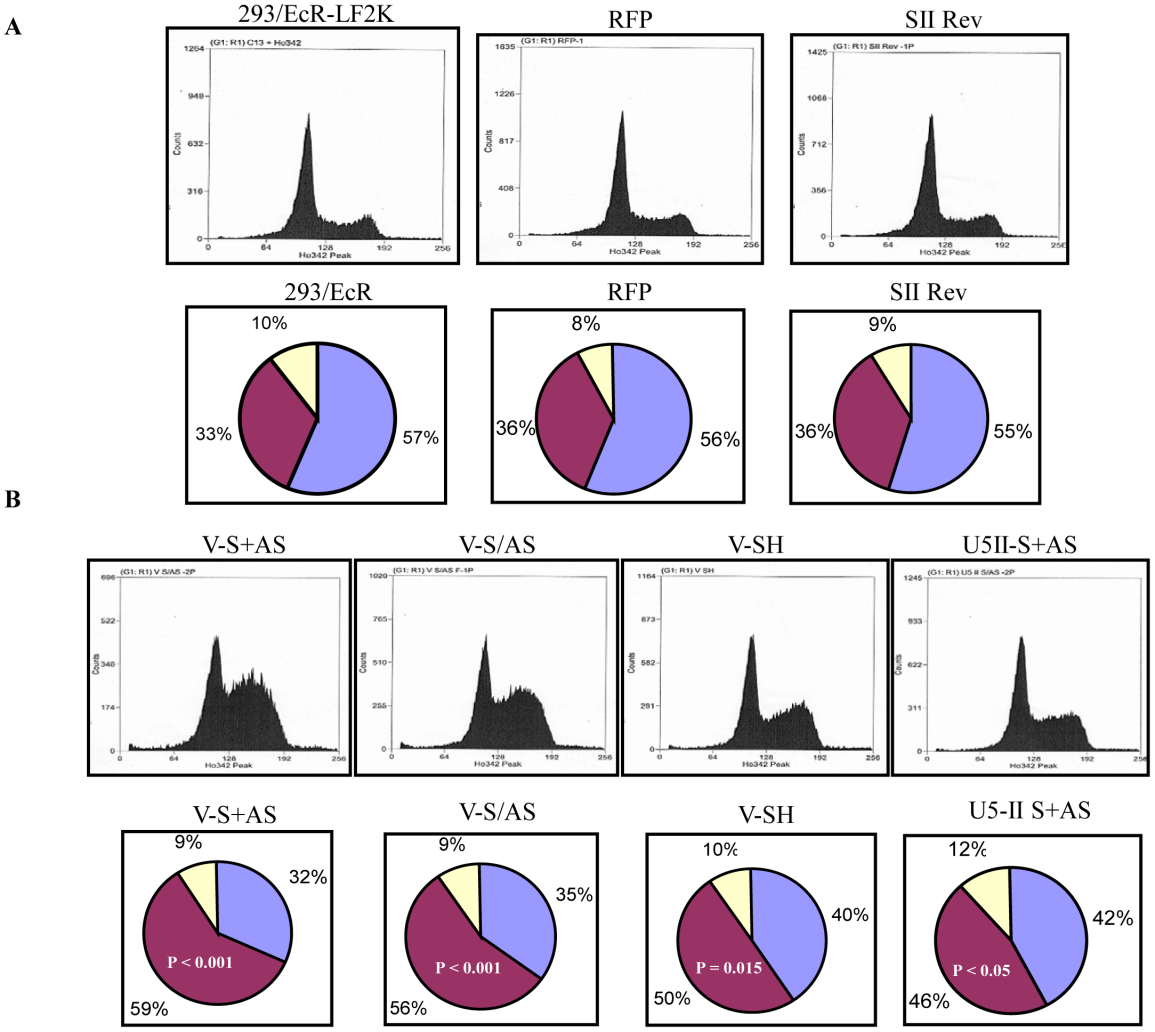
Cell cycle analyses of shRNA treated cells. A) The upper panel (left to right) shows the 293/EcR cells mock transfected with lipofectamine 2000, transfected with DsRed2 plasmid and the irrelevant anti-Rev shRNA. The cell cycle distribution for these treatments was similar to untreated HEK 293 cells. The lower panel illustrates the cell cycle distribution of the control experiments. The 293/EcR mock transfected, DsRED2 plasmid transfected and irrelevant anti-Rev shRNA transfected cells have similar cell cycle distribution patterns to HEK293 cells. B) The upper panel (left to right) demonstrates the cell cycle distribution for 293/EcR cells transfected with anti-U5-200kD siRNA (2 plasmids), sense and antisense cassettes in one plasmid, the same site in short hairpin (shRNA) form as well as a second anti-U5-200kD siRNA (2 plasmid) targeting a different sequence in the U5-200kD mRNA. The V-S+AS, V-S/AS and V-Sh each encode siRNAs targeting the same anti-U5-200 kDa site but are expressed either as separate sense/antisense strands from two plasmids or a single plasmid, or as an shRNA. All three cell cycle distributions illustrate severe S phase arrest due to the knockdown of the U5-200kD target mRNA. The U5II-S+AS site confirms that the cell cycle distribution obtained from the first site is truly due to reduction of the U5-200kD helicase. The lower panel represents the cell cycle distribution of the U5-200kD specific siRNA and shRNA transfected cells. Compared to the control panel A, the percentage of cells in S phase has increased dramatically from 33–36% to 50–59% while the G2/M phase of the cell cycle remained unchanged.

In contrast to the M11 expressing cells, the shRNA site V treated cell lines demonstrated a severe slow growth phenotype. As described above, we analyzed the defect at the level of cell cycle. In the parental HEK 293 cells the distribution of the cell cycle phases is approximately 55–58% G1, 33–37% S and 8–12% G2/M (data not presented). Our experimental controls, mock transfected, DsRED2, and irrelevant shRNA transfected HEK293/EcR cells exhibited the same cell cycle distribution as the HEK293 cells (Fig. 8A). In contrast, the short hairpin targeting site V and the S/AS targeting sites V and II expressing cells exhibited strong cell cycle defects (Fig. 8B). Relative to the control cells, the G1 phase of the cell cycle for the short hairpin RNA and S/AS expressing cells was reduced to 40% and 35%, respectively (Fig. 8B lower panel) and the S phase was concomitantly increased to 50% and 59%, respectively (Fig. 8B lower panel). The G2/M phase of the cell cycle remained unchanged for both treatments (Fig. 8B lower panel). These experiments were repeated eight times, and the average numbers are reported. The cell cycle analyses clearly indicate that the effects of expressing a dominant negative mutant versus an RNAi mediated knockdown of the endogenous gene result in quite different cell cycle perturbations. This marked difference holds true for the effects of these two approaches on the splicing of alpha-tubulin as well.

## Discussion

The RNA helicase Prp44p (also designated SNRNP200, ASCC3L1, HELIC2, Brr2p and Snu246p) has been extensively studied in yeast [5], [6], [8], [9], [18]. Given the important role of this enzyme in the function of the U4/U5/U6 tri snRNP, it is not surprising that mutant analyses have demonstrated that this helicase is an essential protein for cell viability in yeast. *In vitro* splicing analyses of the human homologue, the U5-200kD protein have revealed that this enzyme is an integral part of the spliceosome and is involved in the functional activation of the tri-snRNP [10]. Considering that the *in vitro* splicing studies did not sufficiently explore the other possible role(s) of the helicase in the regulation of cell biology, we wished to extend our studies of this helicase to its intracellular functions. As tools for our analyses, we created the same mutation (domain I GKT-DNT) in the U5-200kD protein that was shown to create a dominant negative mutant in yeast [9]. In addition, we used RNAi to knock down the levels of this protein to study the effects on cell splicing and cell viability.

Mutational analysis of the first helicase motif within domain I surprisingly resulted in only modest splicing defects concomitant with a G2/M delay in human HEK293 cells. RNAi mediated knockdown of the U5-200kD transcript resulted in even less severe splicing defects, but a more severe growth defect phenotype, which appears to be largely a consequence of disruptions in the S phase of the cell cycle. Interestingly, unlike the other splicing factors studied in mammalian cells, disruption of the function of the U5-200kD in HEK293 cells did not lead to apoptotic cell death.

To date, the published reports on the function of this helicase have been limited to mammalian splicing extracts. Functional analysis of the U5-200kD has been previously investigated in HeLa cell splicing extracts using anti-U5-200kD antibodies [5]. The downside of such analyses is that the true biological roles of each domain of the helicase within the cell may be overlooked. Hence, we took advantage of the high homology between the domain I of the yeast and human proteins and created the identical mutation within the U5-200kD gene that created a dominant negative phenotype in yeast [9]. Additionally, through application of RNAi we investigated the biological consequences of knocking down the levels of this RNA helicase in cell culture. Transient overexpression and stable incorporation of this mutant in 293/EcR cells marginally impacted pre-mRNA splicing, as revealed by the slight reduction in alpha-tubulin mRNA and the accompanying increase in the pre-mRNA. The domain I dominant negative mutant form of the U5-200kD is in the first GKT (Walker motif) of the amino terminal RNA helicase domain. This mutation in the yeast homologue perturbed ATP binding, thereby impacting ATPase function and helicase activity. Analogous to the yeast mutant, the incorporation of the U5-200kD dominant negative mutant into a tri-snRNP particle and the spliceosome should similarly affect formation of an active spliceosome in a sub-population of complexes in human cells. In concordance with the minor splicing defect that we observed in the 293/ECR cells, biochemical analysis of the splicing extracts from cells expressing the dominant negative mutant did not show defects in spliceosome assembly or the splicing reaction itself (data not presented).

In yeast, alpha-tubulin pre-mRNA splicing has been shown to be sensitive to mutational perturbation of some splicing factors [11], [19], [20]. Additionally, a growth defect phenotype in yeast has been pinpointed to a G2/M arrest in the case of some splicing factors and S phase arrest in the case of prp17 [20], [21]. Interestingly, the mutation within the first GKT motif of the U5-200kD also resulted in a G2/M stall in 293/EcR cells. At this time, we do not know whether the defect caused by the dominant negative mutant is a consequence of disturbing the splicing of solely the alpha-tubulin pre-mRNA, a single, key cell-cycle sensing protein, or due to a more global defect in splicing. Furthermore, the splicing consensus sequences may play a role in the assembly and kinetics of the spliceosome within cells. By reducing the level of splicing via the dominant negative mutant, pre-mRNAs that have weak splicing regulatory elements might be more strongly affected than those with strong splicing signals. In yeast pre-mRNA splicing mutations in core spliceosomal components revealed that the efficiency of pre-mRNA splicing may be dependent upon the composition of the spliceosome as well as the particular transcript upon which it assembles [22]. Alternatively, the U5-200kD RNA helicase may have other roles in the biology and biogenesis of the cell, for example, a direct role in regulation of the cell cycle which is perturbed by a mutation in the helicase domain I.

Interestingly, the RNAi mediated knockdown of the endogenous U5-200kD mRNA and protein resulted in somewhat different splicing and growth defects. The perturbation of splicing of the alpha-tubulin transcript was not as marked as that observed with the dominant negative mutant. However, cell proliferation and the cell cycle profile were more dramatically affected when compared to the dominant negative mutant studies. The cell cycle analyses clearly indicated that the transfected cells were affected at S phase. Unlike the stable dominant negative expressing cells, the short hairpin transfected cells showed no observable perterbations in the G2/M phase of the cell cycle. One possibility for these discrepant effects is that the abrupt reduction in splicing mediated by U5-200kD knockdown disrupts the stoichiometry of spliceosomal components, which is sensed by a key protein or a complex of S phase specific protein(s). In contrast, formation (assembly) of defective spliceosmes by the domain I dominant negative mutant has a mechanical and therefore more global effect on splicing, resulting in activation of the G2/M checkpoint. Alternatively, the ATPase domains or the domain II of this helicase may play a direct role in regulation of cell cycle progression.

Interestingly, each of the approaches used in this study to interfere with the function of the U5-200kD protein had differential splicing and cell cycle effects in the absence of cell death. The G2/M delay due to the expression of the domain I mutant could be the consequence of a sensing mechanism for monitoring splicing efficiency of the G2/M specific messages within cells. The macular degenerative disease retinitis pigmentosa is associated with mutations in core components of the splicesome, such as the human homologue of prp8. The mutations associated with this disease may cause defects in splicing of a subset of transcripts specific for retinal function [23]–[25]. Furthermore, missense mutations affecting highly conserved codons as well as the mutation between the conserved helicase domains in U5-200kD have been reported in 96 unrelated North American patients and in four generations of Chinese families with autosomal-dominant retinitis pigmentosa (adRP) [26], [27]. It is also noteworthy that a siRNA library based search in HeLa cells to identify genes involved in regulating cell division revealed 37 genes, seven of which were splicing factors [3]. Knockdown of each of the splicing factors resulted in abnormal spindle formation and several of these resulted in cytokinesis defects, but none resulted in cell death. Additionally, a screen for alternative splicing factors revealed that alternative splicing regulation is coupled to cell cycle control. This splicing regulation preceded mitotic arrest, indicating accumulation of pro-death factors in anticipation of G2/M and/or mitotic spindle checkpoints. This splicing regulation likely extends to many coregulated mRNAs including the U5-200kD [28]. The results we obtained from the overexpression of the domain I dominant negative mutant is in agreement with the findings of Kittler, et al. and Pleiss, et al [3], [22]. However, our RNAi mediated data is unique and the phenotype is strikingly different from those thus far reported for splicing factor knockdowns. Our results suggest that in mammalian cell culture at the level of cell cycle progression, splicing may have a regulatory role.

We demonstrated for the first time that overexpression of the mutant core mammalian U5 snRNP and tri-snRNP splicing factor and RNAi-mediated knockdown of this factor have a differential effect in mammalian cells. Further mutational analysis of the second helicase domain and overexpression of the double (domain I and domain II) mutant will reveal whether the U5-200kD has a role in regulation of the cell cycle as well. The simple hypothesis that a splicing defect affects all the pre-mRNAs equally is in disagreement with our findings, which suggest that the spliceosome has the capacity to differentiate among the pre-mRNAs during the cell cycle. Additionally, work with other splicing helicases in cell culture should lead to greater insights into how mammalian cells sense and respond to perturbations of pre-mRNA splicing.

## Materials and Methods

### Ethics Statement

Ethics Committee Approval: No ethics committee approvals such as IRB or IACUC were required for these studies since all the experiments were conducted using established cell lines. There were no human subjects or human clinical samples and no animals used in this study rendering them exempt from ethics committee approval.

### Construction of the Ecdysone-inducible 293/EcR Cell Line (C13)

The HEK 293 cells were grown and maintained in DMEM medium containing 10% fetal bovine serum (FBS). The cells at approximately 60 percent confluency were transfected with the VgRXR plasmid. At 24 hours post transfection the transfection solution was changed to fresh complete media. Two days post transfection the cells were split one to five and kept in the same medium containing zeocin. The cells were maintained in the zeocin containing media until all the untransfected cells disappeared. The plasmid containing cells were reseeded at 1000 cells per plate and maintained in the zeocin-containing medium. The media was replaced every 2 days until the isolated colonies began rising. The grown isolated colonies were transferred to a separate dish and maintained until confluency.

### PCR Mutagenesis of the U5-200kD Gene

In order to test the function and effect of the U5-200kD gene *in vivo,* we created a mutant version of the cDNA using site-directed mutagenesis. We used the QuikChange Site-Directed Mutagenesis kit from the Stratagene Company. This strategy can be used to make point mutations, switch amino acids and delete or insert amino acids. The QuikChange site-directed mutagenesis is carried out using the high fidelity, low processivity *pfu* polymerase to amplify the super-coiled, double-stranded plasmid containing the cDNA insert. The amplification is achieved by the utilization of the two synthetic oligonucleotides containing the desired mutations. The small amount of starting DNA template required to perform this method, the high fidelity of the *pfu* DNA polymerase and the low cycle number all contribute to the high mutation efficiency and decreased potential for random mutation during the amplification reaction. Two synthetic oligonucleotide primers containing the desired mutations were designed based on the U5-200kD cDNA sequence. The oligonucleotide primers each complementary to opposite strand of the recombinant vector containing the cDNA was designed to contain the mutant nucleotides in the middle of the primers. The 30 nucleotide Mut_P_1 primer ^5′^CTACTGGTGCTGATAATACCAACGTGGCCC ^3′^ and Mut_P_ 2 primer ^5′^ GGGCCACGTTGGTATTATCAGCACCAGTAG ^3′^ contain the mutant nucleotides (red). Ten nanograms of the BlueScript KS^+^ plasmid containing the U5-200kD cDNA was amplified using 20 picomole of each Mut primer, 200 nM dNTP, 1×*pfu* specific buffer and 2.5 units of the polymerase. The temperature cycling parameters were 95°C 1 minute, 20 cycles of 95°C 1 minute, 56°C 1 minute and 68°C for 27 minutes, one cycle of 68°C for 10 minutes. The resulting product was digested with Dpn I to remove the parental DNA template. The nicked vector DNA harboring the mutant U5-200kD cDNA was transformed into XL1-Blue competent cells. Plasmids from several transformants were isolated and analyzed by sequencing. The sequence data confirmed the presence of the mutant cDNA in the pBlueScript plasmid.

### Construction of Ecdysone-inducible Mutant U5-200kD Plasmid

The mutant U5-200kD clone 11 (M11) and the pSL1180 plasmids were digested with Not 1 and Xho 1 restriction endonucleases using the New England Biolab buffer 3. The 6.7 kb fragment, mutant U5-200kD, and the 3.4 kb psl1180 fragments were gel purified with QIAquick Gel Extraction Kit (Qiagen) and ligated overnight with T4 DNA Ligase (Promega) according to manufacturer’s protocol. After confirming the recombinant plasmid by restriction digest analysis, the mutant cDNA was released using Sac II and Xho I enzymes and ligated to the pIND plasmid (Invitrogen) containing the Not I and Sac II 5′ untranslated region of the mutant U5-200kD cDNA. The presence of the full-length cDNA was confirmed by digesting pIND-M11 with Not I and Xho I as well as Nhe I and Xho I restriction endonucleases. Additionally, the sequence content of the mutant gene was further confirmed by sequencing.

### Transient Transfection and Expression Analysis of the Inducible Mutant U5-200kD Gene into C13 Cell Line

The pI-M11 plasmid was transiently transfected into the C13 cell line with the Lipofectamine reagent. Twenty-four hours post transfection 4.0 µM of Ponasterone A was added to one of the plates. Twenty-four hours post induction total RNA was isolated from both plates and treated with DNase I enzyme to remove genomic DNA. The DNA-free RNA from each plate was amplified using RT-PCR. The ninety-six microliter annealing reactions were set up using 10 µl of the 10X Taq polymerase buffer, 200 nM dNTP, 40 pmoles of the U5-200kD upstream primer ^5′^caactgcttccagtggaaa^3′^, 40 pmoles of the 3′ mutant specific primer ^5′^agggccacgttggtattat^3′^ and water. The annealing reactions were heated for 1 minute at 80°C and cooled down slowly to 26°C. Each reaction was split into two tubes, and to one tube 6 units of AMV reverse transcriptase enzyme was added and the reaction volume was raised to 49 microliters with water. Water was added to the second half of the reaction up to 49 microliters. Both halves of each reaction were incubated at 37°C for 8 minutes to complete the first strand synthesis. Subsequently, 2.5 units of Taq DNA polymerase were added to all the tubes. The cycling parameters for the PCR reactions were 95°C 5 minutes, 28 cycles of 94°C 1 minute, 58°C 1 minute and 72°C 1 minute, final cycle of 58°C annealing for 1 minute and 72°C extension for 3 minutes terminated the PCR reaction. The products were analyzed on a 1.8% SeaKem Agarose gel.

### Construction of the Ecdysone-inducible Stable Mutant U5-200kD Cell Lines (SMC)

The 293/EcR stable clone was cultured in a 100 mm tissue culture plate and maintained until 90% confluency. The recombinant plasmid pI-M11 containing the mutant form of the U5-200kD gene was packaged with Lipofectamine 2000 and transfected onto the stably expressing transactivating cell line, 293/EcR. Forty-eight hours post transfection the cells were diluted into five plates and fed with the DMEM media containing 10% FBS, penicillin/streptomycin, glutamine, sodium pyruvate, 400 µg/ml zeocin and 700 µg/ml G418. The media was changed every two days until the untransfected cells were dead. The surviving cells were grown to 70% confluency and passed twice. In order to clone individual cells, one thousand cells were seeded in a 10cm^2^ plate and maintained until individual colonies began rising. Several individual colonies of different sizes were picked and transferred into six well dishes. The colonies were maintained and froze in freezing media, 90% FBS and 10% DMSO.

### Analysis of the Stably Expressing Ecdysone-inducible Mutant U5-200kD Clones

Equal numbers of the 293/EcR and different stable mutant clones were seeded duplicate plates on day zero. At twenty-four, forty-eight and seventy-two hours post seeding one dish for each cell line received fresh media containing three micromolar Ponasterone A (induced). The other dish for each cell line only received fresh medium (uninduced). On the fourth day cells were harvested and counted by the hemocytometer. The total cell number for each cell line was calculated and graphed using Excel.

### Detection of the Mutant U5-200kD mRNA from the SMC10 Clone

Total cellular RNA was extracted from various cell lines using RNA Stat60 according to the manufacturer’s protocol. Mutant U5-200kD mRNA was detected by using allele specific RT-PCR and mutant specific primers. Initially, a gene-specific oligonucleotide, U5-413, ^5′^CATCCACATTGAT ^3′^was designed to perform the reverse transcription step and to synthesize the cDNA. For each cell line, a reaction mixture containing total cellular RNA, forty picomoles of the U5-413 reverse primer, 200 µM dNTP (Roche) and 10X Taq polymerase buffer (Eppendorf) in a final volume of ninety-four µl was heated at 80°C for 2 minutes and slowly cooled down to room temperature. The annealed oligonucleotide-RNA mix was divided equally into two PCR tubes. The annealed oligonucleotide was extended by AMV reverse transcriptase enzyme to synthesize the first strand, cDNA. The synthesized cDNA was further amplified by using 20 picomoles of each forward primer M5-4 ^5′^AGGTTCTGGACTTGGA^3′^ and the mutant specific reverse primer ^5′^GGCCACGTTGGTATTA^3′^ (bold letters represent mutant bases) using 1.5 units Taq DNA polymerase in a final volume of fifty µl. The mutant reverse primer is designed for the detection of mutant U5-200kD and ends with mutant specific bases at the 3′ terminus. These primers can in a temperature sensitive manner avoid the wild type endogenous U5-200kD mRNA and detect the mutant U5-200kD mRNA from the SMC10 cells. The cycling parameters were one cycle of 95°C for five minutes to inactivate the AMV reverse transcriptase, thirty-three cycles of 56°C annealing for one minute, 72°C extension for one minute and 94°C denaturation for one minute. Finally, a single cycle consisting of 56°C annealing for one minute and 72°C extension for three minute was applied to complete the extension. The minus reverse transcriptase reactions were treated equally as the experimental samples. The RT-PCR reactions were analyzed in a 1.8% agarose gel.

### Detection of Alpha-tubulin mRNA from the 293/EcR and SMC10 Cell Lines

Reaction mixtures containing total cellular RNA from each cell line, 40 picomoles of each forward, αTF3, (^5′^GAATGCATCTCAGTCCACGT^3′^) and reverse, αTR3, (^5′^CAGCACTGGGTCAATGATG^3′^) gene-specific primers, 200 µmolar dNTP and 10X Taq polymerase buffer were assembled in final volume of ninety-six µl. These reactions were heated at 80°C for two minutes and slowly cooled down to room temperature. The annealed oligonucleotide-RNA reactions were divided equally into two PCR tubes. The AMV reverse transcriptase enzyme was added to one tube of each duplicate reaction to synthesize the first strand or cDNA. The reactions were incubated at 37°C for eight minutes and cooled down to 4°C. The synthesized cDNA was further amplified by addition of 1.5 units of Taq DNA polymerase in the final volume of fifty µl. The cycling program used was as follows: one cycle of 95°C for five minutes to inactivate the reverse transcriptase, thirty-two cycles of 61°C annealing for one minute, 72°C extension for thirty seconds and one minute 94°C denaturation. A single cycle consisting of one minute at 61°C and three minutes at 72°C was applied to complete the PCR reaction. The second duplicate reaction was treated equally in the absence of the reverse transcriptase enzyme. The products of the reactions were resolved on a 1.8% agarose gel.

### Detection of Alpha-tubulin Pre-mRNA from the 293/EcR and SMC10 Cell Lines

A fifteen nucleotide gene specific RT primer, αT3’EiJ (^5′^ATCTCATCTGGAAGG^3′^) was used to carry out the first strand synthesis. The oligonucleotide-RNA annealing reactions were assembled using total cellular RNA, forty µM of αT3’EiJ primer, 200 µM of dNTP and 10X Taq DNA polymerase buffer in a final volume of ninety-four µl. These reactions were heated for 2 minutes at 80°C and slowly cooled down to 40°C. The mixture was divided equally into two PCR tubes. To one tube AMV reverse transcriptase enzyme was added to synthesize the first strand and to other tube equivalent volume of water was added. Both tubes were incubated at 37°C for 8 minutes and then cooled down to 4°C. Both tubes were subjected to PCR amplification in presence of 20 picomoles of each forward αTEiJ (^5′^CGGTCATTGGTGAGAGGAA^3′^), reverse IαT (^5′^ AACCACAAAGCTATGCTCAG ^3′^) primers and 1.5 units of Taq DNA polymerase in a final volume of fifty µl. The PCR program used consisted of a single cycle at 95°C for five minutes to inactivate the AMV reverse transcriptase, thirty-nine cycle of one minute annealing at 63°C, thirty seconds extension at 72°C and one minute denaturation at 94°C. The reactions were ended with a single cycle of one minute annealing at 63°C and three minutes extension at 72°C. The RT-PCR products were resolved on a 2% agarose gel.

### Cell Cycle Analysis of the 293/EcR and the SMC10 Cell Lines

On day zero an equal number of cells for each cell line were seeded in duplicate 100 mm tissue culture dishes (Falcon). Twenty-four, forty-eight and seventy-two hours post seeding growth media containing Ponasterone A was added to one dish and the other dish received an equal volume of growth medium only. On the fourth day cells were harvested and washed with PBS. The cell pellet was resuspended in PBS, and then cold 100% ethanol was added dropwise while gently vortexing the cell suspension. The samples were stored at –20°C overnight. The next day fixed cells were centrifuged and pelleted. The cell pack was washed twice with PBS and resuspended in PBS. The permeablized cells were treated with RNase A and incubated at 37°C for half an hour. The mixture was centrifuged, and the supernatant was decanted. The cell pack was resuspended in PBS, and propidium iodide solution was added to a final concentration of 100 µl/ml. The cell suspension was incubated at room temperature in the dark for approximately one hour. Finally, the samples were subjected to flow cytometry for cell cycle analysis.

### Construction of the PolIII siRNA Expression Cassettes

The U5-200kD specific siRNA expression cassettes were constructed using U6+1 polIII promoter and a string of 4–6 Ts as termination signal. PolIII expression cassettes encoding the sense and antisense siRNA were constructed and cloned in tandem in PCR2.1 plasmid using polymerase chain reaction. The forward primer U6NSB (^5′^ ATAAGAATGCGGCCGCCCCGGGGATCCAAGGTCGGG ^3′^) complementary to 5′ end of the U6 promoter (bold letters) and the reverse primers S5S (^5′^ AGATCTAAAAA *GGTTCACTAGCACATGGTATC*GGTGTTTCGTCCTTTCCAC ^3′^ ) or S5AS (^5′^AGATCTAAAAA*GATACCATGTGCTAGTGAACCGGTGTTTCG TCCTTTCCAC* ^3′^) complementary to the 3′ end of the U6 promoter harboring the sense and antisense sequences (italic letters) of the siRNA gene respectively were used to generate the PCR-based polIII expression cassettes. For each of the cassettes the hot start PCR reaction mixture was set up using ampliwax bead (Perkin Elmer). The lower phase of the reaction consist of 10X Taq polymerase buffer, 200 µM dNTP (Roche) and 10 µM of U6NSB and 10 µM of either S5S or S5AS primers in a final volume of 25 µl. Samples were heated at 80°C for five minutes, 24°C for one minute and cooled down to 4°C for two minutes. The upper phase of the reaction consist of 10X Taq buffer, 10 ng of pTZU6+1 plasmid and 1 unit of Taq DNA polymerase in total volume of 75 µl added on top of the solidified wax barrier. The amplification program was as follows: one cycle of 95°C for five minutes, twenty-eight cycles of 55°C annealing temperature for one minute, 72°C extension temperature for one minute and 94°C denaturation for one minute. A final cycle consisting of 55°C for one minute and 72°C for seven minutes was applied to terminate the reaction. The PCR product was gel purified using the Bio 101 gel extraction kit (Bio 101) according to manufacturer’s protocol. The purified product was then cloned into PCR2.1 (Invitrogen) using the TA cloning method. The isolated clones were analyzed and confirmed by EcoR1 (New England Biolabs) restriction endonuclease digestion and later by sequencing. In order to clone both sense and antisense cassettes in one plasmid, the siRNA antisense cassette was released from the PCR2.1-S5AS plasmid using Spe1 and ApaI enzymes and sub-cloned into PCR2.1-S5S plasmid linearized with Xba1 and Apa1 enzymes. The presence of both cassettes was confirmed by restriction endonuclease analysis using Spe1 and Apa1 enzymes.

### Construction of the PolIII shRNA Expression Plasmids

The polIII short hairpin expression plasmids were constructed applying a PCR-based approach. An oligonucleotide, SHU5, reverse primer (^5′^ TCTAGAAAAA GATACCATGTGCTAGTGAACC*TGACAGGAAG*GGTTCACTA GCACATGGTATCGGTG ^3′^), containing the sequences complementary to the 3′ end of the U6 promoter, sense siRNA (bold letters), mir23 loop (italic letters) antisense siRNA (bold letters) and the string of As was synthesized and used in a hot start PCR reaction with the U6NSB forward primer. The reaction contained 10 µM of each primer, Taq DNA polymerase buffer, and 200 µM dNTPs in a volume of 25 µl. The reaction mix was overlaid with an ampliwax bead and subjected to 80°C heating for five minutes, 24°C for one minute and cooled down to 4°C for two minutes. The top phase of the reaction consists of Taq DNA polymerase buffer 10 nanograms of the purified S5S fragment previously digested with DraIII and HindIII restriction enzymes (New England Biolabs) and 1.5 units of Taq DNA polymerase in a volume of 75 µl. The amplification program used was as follows: one cycle of 95°C for five minutes to denature the AMV reverse transcriptase, twenty-eight cycles of 55°C annealing, 72°C extension and 94°C denaturation for one minute each. A final cycle of 55°C annealing for one minute and 72°C extension for three minutes was performed to terminate the reaction. The polIII short hairpin cassette was gel purified and cloned into the PCR2.1 plasmid by TA cloning. The recombinant plasmids were confirmed by EcoR1 digestion to release the polIII cassette and by sequencing to confirm the nucleotide sequence of elements within the cassette.

### RT-PCR Detection of the Histone 2A (H2A) mRNA

RT –PCR reactions containing total cellular RNA, 40 µM H2A-F (^5′^ CACTTTCTGACTTAGGCC ^3′^), 40 µM H2A-R (^5′^ AAGTTCAGCCCTTACTTGC ^3′^), Taq DNA polymerase buffer, final concentration of 1X and 200 µM dNTP in a volume of 96 µl were assembled. The reaction mixture was heated at 80°C for 2 minutes and slowly cooled down to room temperature. The annealed oligonucleotide-RNA mixtures were divided equally into two PCR tubes. To one tube 7 units of AMV reverse transcriptase enzyme was added and the other tube received an equal volume of water. Both positive and negative RT reactions were incubated at 37°C for 8 minutes and cooled down to 4°C. The synthesized cDNA and the negative RT reactions were further amplified by addition of one and half units of Taq DNA polymerase to each tube. The amplification program comprised of one cycle of 95°C for 5 minutes to denature the AMV reverse transcriptase, 21 cycles of 52°C annealing, 72°C extension and 94°C denaturation for one minute each. A final cycle of 52°C annealing for one minute and 72°C extension for 3 minutes was performed to complete the amplification reaction. The PCR product was resolved on a 1.8% agarose gel.

### RT-PCR Detection of the Wild Type U5-200kD mRNA

Total cellular RNA, 40 µM of the U5F2 (^5′^ GGCTTCAACATCAGCCAT ^3′^) and U5R2 (^5′^ CTGAGCTGAAGAGCTGCT ^3′^) primers, 200 µM of dNTP and Taq DNA polymerase buffer were assembled in a final volume of 96 µl. The U5R2 primer was annealed to the U5-200kD mRNA by heating the mixture at 80°C for two minutes and slowly cooling down to room temperature. The annealed RNA-oligonucleotide mix was split into two PCR tubes. One tube was used for positive RT and the other for the negative RT reactions. The AMV reverse transcriptase enzyme was added to the positive RT reaction while the negative reaction received an equivalent volume of water. Both reactions were incubated at 37°C for eight minutes and cooled down to 4°C. Both reactions were further amplified by the addition of 1.5 units of Taq DNA polymerase in a final volume of fifty µl. The amplification program used consisted of one cycle at 95°C for five minutes to denature the AMV reverse transcriptase, twenty-eight cycles of one minute at 52°C annealing, one minute at 72°C extension and one minute at 94°C denaturation. A final cycle of 52°C annealing for one minute and 72°C extension for three minutes was applied to complete the reaction. The PCR product was resolved on a 1.8% agarose gel.

### RT-PCR Detection of EGFP mRNA

Total cellular RNA was isolated from cells transfected with pIND-int-EGFP and subsequently DNase-treated to remove plasmid DNA. Total cellular RNA combined with 40 picomoles of reverse primer (^5′^ ACTTGAAGAAGTCG ^3′^), 200 µM dNTP and Taq DNA polymerase buffer in a total volume of 94 µl was heated at 80°C for two minutes and slowly cooled down to 39.5°C temperature. The annealed RNA-oligonucleotide mixture was divided into two PCR tubes for further steps. One tube was used for positive RT and the other for the negative RT reactions. The AMV reverse transcriptase enzyme was added to the positive RT reaction while the negative reaction received an equivalent volume of water. Both reactions were incubated at 37°C for eight minutes and cooled down to 4°C. Both reactions were further amplified by the addition of a cocktail consisting of 20 µM 5′EXEX (^5′^ ACTGGGCAGGTGTCCACT ^3′^) primer, 20 µM 3′EGFP (^5′^ TGCCGGTGGTGCAGATGA ^3′^ ) primer and 1.5 units of Taq DNA polymerase in a final volume of fifty µl. The amplification program used consisted of one cycle at 95°C for five minutes to denature the AMV reverse transcriptase, twenty-nine cycles of one minute at 64°C annealing, thirty seconds at 72°C extension and one minute at 94°C denaturation. A final cycle of 64°C annealing for one minute and 72°C extension for three minutes was applied to complete the reaction. Subsequently, the PCR product was analyzed on a 1.8% agarose gel.

### RT-PCR Detection of Intron-EGFP Pre-mRNA

Isolated total cellular RNA from each treatment was mixed with 40 µM of 3′RT2 (^5′^TTGCTCACCATGGT ^3′^) primer, 200 µM dNTP and Taq DNA polymerase buffer in a final volume of 94 µl. The reaction was heated at 80°C for two minutes and slowly cooled down to 39.5°C. The annealed RNA-oligonucleotide mixture was divided into two PCR tubes for further steps. One tube was used for positive RT and the other for the negative RT reactions. The AMV reverse transcriptase enzyme was added to the positive RT reaction while the negative reaction received an equivalent volume of water. Both reactions were incubated at 37°C for eight minutes and cooled down to 4°C. Both reactions were further amplified by the addition of a cocktail consisting of 20 µM 5′EXINT (^5′^ ACTGGGCAGGTAAGTATCA ^3′^) primer, 20 µM 3′Int-E-R (^5′^ TCTAGAG TCGACTCTAGAG ^3′^) primer and 1.5 units of Taq DNA polymerase in a final volume of fifty µl. The amplification program used consists of one cycle denaturation at 95°C for five minutes, thirty-nine cycles of one minute at 62°C annealing, thirty seconds at 72°C extension and one minute at 94°C denaturation. A final cycle of 62°C annealing for one minute and 72°C extension for three minutes was applied to complete the reaction. Subsequently, a semi-nested PCR amplification reaction was performed to enhance the product yield. The second reaction consisted of two µl of the first PCR product as template, 20 µM of 5′EXINT forward primer, 20 µM of nested 3′INTEX (^5′^ GGACACC-TGTGGAGAGAA ^3′^) reverse primer, 200 µM dNTP, Taq DNA polymerase buffer and 1.5 units of Taq DNA polymerase enzyme in a final volume of 50 µl. The second amplification parameters were one cycle of 95°C denaturation followed by six cycles of one minute at 62°C annealing, thirty seconds at 72°C extension and one minute at 94°C denaturation. A final cycle of one minute at 62°C and three minutes at 72°C extension was applied to complete the reaction. The PCR product was analyzed on a 2% agarose gel.

### Detection of Alpha-tubulin Pre-mRNA from 293/EcR Cells Transfected with shRNA Plasmid

Total cellular RNA isolated from transfected cells was DNase-treated to remove the plasmid and genomic DNA content. The DNase-treated RNA was used to set up the RT-PCR reactions. Reaction mixtures containing an adequate amount of RNA, forty µM αT3’EiJ primer, 200 µM dNTP and 10X Taq DNA polymerase buffer in a final volume of ninety-four µl were assembled. These reactions were heated for 2 minutes at 80°C and slowly cooled down to 44°C. The mixture was divided equally into two PCR tubes. To one tube AMV reverse transcriptase enzyme was added to synthesize the first strand and to the other tube an equivalent volume of water was added. Both tubes were incubated at 37°C for 8 minutes and then cooled down to 4°C. Both tubes were subjected to PCR amplification in the presence of 20 picomoles of each forward αTEiJ and reverse IαT primers and 1.5 units of Taq DNA polymerase in a final volume of fifty µl. The PCR program used consisted of a single cycle at 95°C for five minutes to inactivate the AMV reverse transcriptase, thirty-nine cycles of one minute annealing at 64°C, thirty seconds extension at 72°C and one minute denaturation at 94°C. The reactions were ended with a single cycle of one minute annealing at 64°C and three minutes extension at 72°C. Subsequently, a semi-nested PCR amplification reaction was performed to enhance the product yield.

The second reaction consisted of five µl of the first PCR product as template, 20 µM of αTEiJ forward primer, 20 µM of nested A-TN5p (^5′^ ATGCTCAGCAGGGACCTT C ^3′^) reverse primer, 200 µM dNTP, Taq DNA polymerase buffer and 1.5 units of Taq DNA polymerase enzyme in a final volume of 50 µl. The second amplification parameters were one cycle of 95°C denaturation followed by 20 cycles of one minute at 63°C annealing, thirty seconds at 72°C extension and one minute at 94°C denaturation. A final cycle of one minute at 63°C and three minutes at 72°C extension was applied to complete the reaction. The PCR product was analyzed on a 2% agarose gel.

### Transient Transfection of siRNA and shRNA Plasmids in 293/EcR Cells

On day zero an equal number of 293/EcR cells were seeded in the tissue culture dishes. Twenty-four hours post transfection the appropriate shRNA or siRNA plasmids were co-transfected with pIND-int-EGFP splicing reporter plasmid, DsRED2 transfection efficiency plasmid and pCR2.1-U6+1 plasmid as carrier DNA. Seventy-two hours post transfection 3 µM Ponasterone A was added to the dishes to induce expression of the reporter plasmid. Approximately ninety-six hours post transfection cells were harvested and a fraction of them were subjected to flow cytometry for the expression of Red and EGFP proteins. Total cellular RNA was isolated from the remaining cells using RNA Stat60.

### Cell Cycle Analysis of the siRNA and shRNA Transfected Cells

This protocol is predominantly used to analyze the DNA content of live cells.

On day zero an equal number of 293/EcR cells were seeded in 60 mm tissue culture dishes. Twenty-four hours post seeding appropriate plasmids were transfected in the cells. At ninety-six hours post transfection cells were harvested and spun down. The cell packs were resuspended in ten ml of complete media. Two hundred fifty microliters of the cell suspension was diluted two fold in the growth media. Hoechst 33342 staining solution was added to the cell suspension (∼10^6^ cells/ml) to obtain a final dye concentration of 10 µg/ml. The mixture was incubated in the tissue culture incubator for approximately one hour. After the incubation the mixture was subjected to flow cytometry, and the DNA content of the transfected cells was anlayzed.

## Supporting Information

Figure S1 A) RT-PCR analysis of EGFP pre-mRNA and mRNA reporter expression using the site II siRNA (UII S/AS) against U5-200KD mRNA indicated reduction of the EGFP mRNA and accumulation of the EGFP pre-mRNA (upper panel). qRT-PCR analysis indicated reduced expression of the spliced EGFP mRNA after transfection of the UII S/AS siRNA construct (lower panel). B) RT-PCR analysis of alpha-tubulin pre-mRNA and mRNA expression using the site II siRNA against U5-200kd mRNA indicated reduction of the alpha-tubulin mRNA and accumulation of the alpha-tubulin pre-mRNA.
(TIF)
