# Supplementary figures and images for: Cell Cycle Abnormalities Associated with Differential Perturbations of the Human U5 snRNP Associated U5-200kD RNA Helicase
Source: PLoS One. 2013 Apr 29;8(4):e62125. doi: 10.1371/journal.pone.0062125 (PMC3639242; doi:10.1371/journal.pone.0062125)

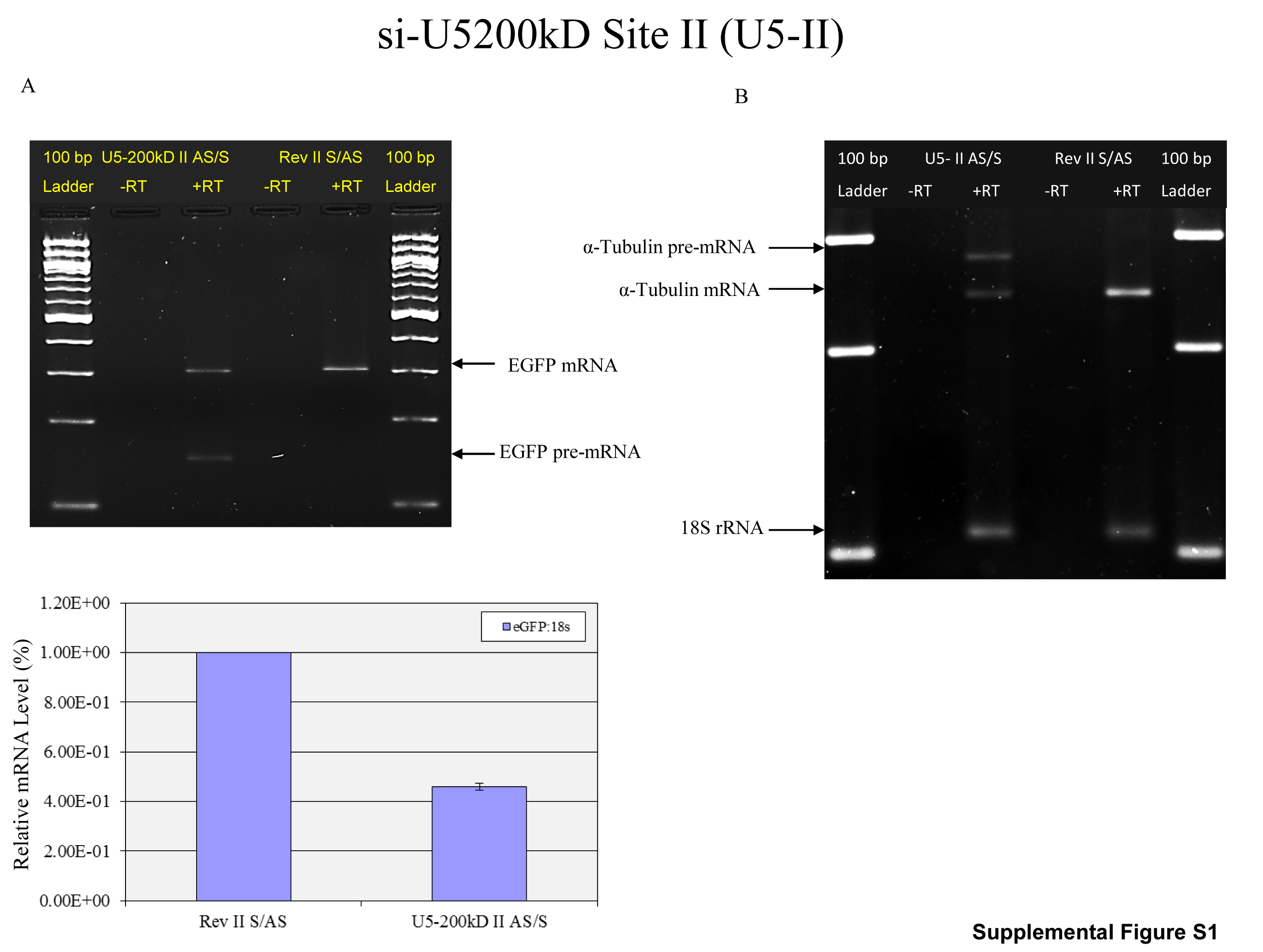

Supplement: Figure S1 — A) RT-PCR analysis of EGFP pre-mRNA and mRNA reporter expression using the site II siRNA (UII S/AS) against U5-200KD mRNA indicated reduction of the EGFP mRNA and accumulation of the EGFP pre-mRNA (upper panel). qRT-PCR analysis indicated reduced expression of the spliced EGFP mRNA after transfection of the UII S/AS siRNA construct (lower panel). B) RT-PCR analysis of alpha-tubulin pre-mRNA and mRNA expression using the site II siRNA against U5-200kd mRNA indicated reduction of the alpha-tubulin mRNA and accumulation of the alpha-tubulin pre-mRNA. (TIF) [file pone.0062125.s001.tif]
